# Identification of Genetic Predisposition in Noncirrhotic Portal Hypertension Patients With Multiple Renal Cysts by Integrated Analysis of Whole-Genome and Single-Cell RNA Sequencing

**DOI:** 10.3389/fgene.2021.775470

**Published:** 2021-11-12

**Authors:** Yanjing Wu, Yongle Wu, Kun Liu, Hui Liu, Shanshan Wang, Jian Huang, Huiguo Ding

**Affiliations:** ^1^ Department of Gastroenterology and Hepatology, Beijing You’an Hospital, Affiliated with Capital Medical University, Beijing, China; ^2^ Department of General Surgery, Beijing Friendship Hospital, Affiliated with Capital Medical University, Beijing, China; ^3^ Department of Pathology, Beijing You’an Hospital, Affiliated with Capital Medical University, Beijing, China; ^4^ Beijing Institute of Hepatology, Beijing You’an Hospital, Affiliated with Capital Medical University, Beijing, China; ^5^ Experimental Center, Beijing Friendship Hospital, Affiliated with Capital Medical University, Beijing, China

**Keywords:** whole-genome sequencing, single-cell RNA sequencing, gene mutations, multiple renal cysts, noncirrhotic portal hypertension

## Abstract

**Background and Aims:** The multiple renal cysts (MRC) occur in some patients with noncirrhotic portal hypertension (NCPH) could be a subset of ciliopathy. However, the potential genetic influencers and/or determinants in NCPH with MRC are largely unknown. The aim of this study was to explore the potential candidate variants/genes associated with those patients.

**Methods:** 8,295 cirrhotic patients with portal hypertension were enrolled in cohort 1 and 267 patients affected with NCPH were included in cohort 2. MRC was defined as at least two cysts in both kidneys within a patient detected by ultrasonography or computed tomography. Whole-genome sequencing (WGS) was performed in nine patients (four from cohort 1 and five from cohort 2). Then we integrated WGS and publicly available single-cell RNA sequencing (scRNA-seq) to prioritize potential candidate genes. Genes co-expressed with known pathogenic genes within same cell types were likely associated NCPH with MRC.

**Results:** The prevalence of MRC in NCPH patients (19.5%, 52/267) was significantly higher than cirrhotic patients (6.2%, 513/8,295). Further, the clinical characteristics of NCPH patients with MRC were distinguishable from cirrhotic patients, including late-onset, more prominent portal hypertension however having preserved liver functions. In the nine whole genome sequenced patients, we identified three patients with early onset harboring compound rare putative pathogenic variants in the known disease gene *PKHD1*. For the remaining patients, by assessing cilia genes profile in kidney and liver scRNA-seq data, we identified *CRB3* was the most co-expressed gene with *PKHD1* that highly expressed in ureteric bud cell, kidney stromal cell and hepatoblasts. Moreover, we found a homozygous variant, *CRB3* p.P114L, that caused conformational changes in the evolutional conserved domain, which may associate with NCPH with MRC.

**Conclusion:** ScRNA-seq enables unravelling cell heterogeneity with cell specific gene expression across multiple tissues. With the boosting public accessible scRNA-seq data, we believe our proposed analytical strategy would effectively help disease risk gene identification.

## Introduction

Cirrhotic portal hypertension complicated esophageal-gastric variceal bleeding (EGVB), ascites, hepatic encephalopathy (HE), acute kidney injury (AKI) or hepatorenal syndrome (HRS) and splenomegaly accompanied by severe liver disfunctions, is almost accounted for 80–85%. In clinical practice, a small number of patients present with portal hypertension, such as splenomegaly and or EGVB, HE, their clinical manifestations are similar to the liver cirrhosis,but in fact, these patients do not have liver cirrhosis, that is non-cirrhotic portal hypertension (NCPH) (Khanna and Sarin, 2019; Gao et al., 2020). Currently, chronic infections, autoimmune disorders, and genetic determinants have been reported to be associated with pathogenesis of NCPH (Vilarinho et al., 2016; Wu et al., 2019). Interestingly, the NCPH is common in cystic fibrosis-associated liver disease (Boëlle et al., 2019). Therefore, we speculate that liver disease in multiple renal cysts (MRC) present as NCPH, except for a subset of patients with ciliopathy affected by hepatorenal fibrocystic diseases (HFDs), such as autosomal recessive polycystic kidney disease (ARPKD) or Caroli syndrome (Abdul Majeed et al., 2020; McConnachie et al., 2021). HFDs are a group of ciliopathies and genetic disorders that involve developmental abnormalities in the portobiliary system in association with fibrocystic degeneration of the kidney (Lasagni et al., 2021). HFDs can cause enlarged kidneys, cyst formation, biliary duct dilation, and congenital hepatic fibrosis (CHF), resulting in portal hypertension (Myram et al., 2021). Therefore, patients with NCPH with MRC may be a non-classical genetic mutations or HFD phenotype.

Currently, single-cell RNA sequencing (scRNA-seq) has revolutionized developmental biology and genomics. ScRNA-seq is a powerful tool that can be used to elucidate the cellular composition in the interest tissue, to define undescribed rare cell subsets, to dissect regulators controlling cell fate transition, to pinpoint cell-type specific responses to stress or stimulation, and to identify mechanisms of cell-cell crosstalk (Han et al., 2020). In this study, we applied a novel analytic approach that integrated whole-genome sequencing (WGS) and scRNA-seq data to survey potential genetic modifiers or candidate disease genes in NCPH patients with MRC.

## Methods

### Study Design and Patients

Total 8,295 cirrhotic portal hypertensive patients, diagnosed by medical history, signs and imaging of portal hypertension according to chinese guidelines on the management of liver cirrhosis (Xu et al., 2020), were enrolled in cohort 1. The etiologies of liver cirrhosis included hepatitis B (65.04%), hepatitis C (15.79%), alcoholic cirrhosis (12.35%), and autoimmune liver disease (6.82%). Two hundred sixty-seven patients with NCPH in cohort 2, confirmed by radiologists, hepatologists and pathologists based on enhanced computed tomography (CT) or nuclear magnetic resonance imaging (MRI) and/or pathology according to guidelines on the management of NCPH of EASL (Khanna and Sarin, 2014). The signs and symptoms, laboratory and endoscopy data were obtained from Electronic Medical Record Management System (EMRS). The portal and splenic vein diameter and splenic thickness were measured using abdominal ultrasonography. Model for end-stage liver disease (MELD) and Child-Pugh score were assessed for the severity of cirrhosis. Transient elastography liver stiffness measurement (LSM) was performed using FibroScan™ (Echosens, Paris, France). The MRC were defined as more than two cysts in both kidneys detected by ultrasonography or CT.

The study protocol was performed in compliance with the Declaration of Helsinki and approved by the Ethics Committees of Beijing You’an Hospital Capital Medical University. Signed informed consent was obtained from each participant for using samples, materials and publication.

### Whole-Genome DNA Sequencing

The peripheral blood of nine patients with MRC (four from cohort 1 and five from cohort 2 separately) were collected for WGS to explore potential genetic modifiers or candidate disease genes. First, the genomic DNA was extracted from the peripheral blood of those patients. The whole-genome DNA Sequencing libraries were prepared according to the manufacturer’s instructions. The raw reads were produced by a BGISEQ-500 sequencer at an average depth of 40×. The rare putatively pathogenic variants were validated by Sanger sequencing.

### Genomic DNA Analysis

Sequencing data were quality controlled with adapter and aligned to human reference genome build hg19 (http://www.gencodegenes.org/releases/19.html) with BWA aligner (Li and Durbin, 2009). The GATK best practice Haplotype Caller pipeline was implemented for SNV and indel calling (Li et al., 2009; McKenna et al., 2010). SV was called with lumpy software, and CNV was detected with FreeC software (Layer et al., 2014). All SNV variants were annotated using ANNOVAR for bioinformatics analysis (Wang et al., 2010). Several genomic databases, including the 1,000 Genomes (1000G), ExAC (Exome Aggregation Consortium), Exome Sequencing Project (ESP), gnom AD (both WES and WGS databases), and CG46, were used to assess the variant frequency in the population. MCAP, SIFT, Polyphen2-HDIV, Polyphen2-HVAR, MutationTaster, MutationAssessor, and Clinvar were implemented to annotate the effect of missense variants. GERPs were used to evaluate the conservation of the variant locus. Rare putative pathogenic variants were filtered as follows (Gao et al., 2020): the allele frequency of the candidate variant should be lower than 0.01 among 1000G, ExAC, ESP 6500, Genome Aggregation (GA) and Complete Genomics 46 (CG46) Databases (Khanna and Sarin, 2019); amino acid changing variants were kept, and GERP scores should be higher than 2.0 (Vilarinho et al., 2016); truncating variants were kept, and for missense variants, MCAP scores higher than 0.6 were automatically kept, whereas for other variants, the effect should be annotated as “Deleterious” or “Highly pathogenic” by at least two software programs for MCAP scores between 0.025 and 0.6. PKHD1 protein domain prediction was obtained using SMART (Letunic et al., 2021). Cilia genes were obtained from Syscilia.org for downstream analysis.

### Single-Cell RNA Sequencing Analysis

Five sets of scRNA-seq data were used in this study. Summarized gene expression matrices derived from multiple organs from human fetuses were obtained from the GEO database via accession number GSE156793 (Cao et al., 2020). Single-cell expression data from kidneys were generated from mice under accession number GSE140023 (Conway et al., 2020). The adult human kidney and human liver single cell transcriptome was achieved by accession GSE114530 (Hochane et al., 2019), GSE131685 (He et al., 2020) and GSE159929 (Liao et al., 2020), respectively. The single cell RNA sequencing analysis was implemented with Seurat package (Version 3.9.9). Cells were discarded according to the following criteria (Gao et al., 2020): cells that had fewer than 400 genes (UMI >0) (Khanna and Sarin, 2019); cells that had fewer than 600 UMI or over 10,000 UMI; and (Vilarinho et al., 2016) cells that had more than 15% mitochondrial UMI counts. After the above quality control, for mouse kidney, human fetal kidney, human adult kidney and human liver scRNA-seq analysis, we performed log-normalization with the “vst” method and identified 2000, 3,000, 3,000 and 3,000 variable features, respectively. We then scaled by setting the parameter “vars.to.regress” to “percent.mito” and “nCount_RNA”. Principal component analysis (PCA) was performed using the “RunPCA” function. The number of PCs was chosen by visualization plot with the “ElbowPlot” function. A shared nearest neighbor (SNN) graph was constructed using the “FindNeighbors” function with the top 40 PCs, then cells were clustered by the “FindClusters” function with the “resolution” parameter set to 0.5. The “RunUMAP” function was used for the visualization plot with the “umap-learn” method, setting “n.neighbors” to 40L, “dims” to 1:40, and “min.dist” to 0.3. Marker genes for each cluster were detected using the “FindAllMarkers” function, setting the parameter “min.pct” to 0.3 and “logfc.threshold” to 0.6. Subsequently, cell clusters were annotated manually to the major cell types according to known markers. Any cluster with multiple markers of two types of cells was manually discarded as a doublet.

### Protein Conformation Modeling

The full-length human *CRB3* was not annotated in the PDB Database; thus, the intact wild-type and mutated CRB3 protein sequences were annotated by Phyre2 (Kelley et al., 2015). Then, protein conformational alteration was predicted using Chimera software (Pettersen et al., 2004).

### Data Visualization and Statistics

Microsoft R Open (version 3.6.1, https://mran.microsoft.com/) was used. The R packages ggplot 2 (version 3.1.0) and pheatmap (version 1.0.12) were used to generate graphs of the data. Continuous variables were compared using independent T test if data were normally distributed or Mann Whitney U test. The categorical variables were compared using *X* ^2^ tests performed with SPSS 22.0 (IBM, United States).

## Results

### Prevalence and Clinical Characteristics of NCPH Patients With MRC

**FIGURE 1:**
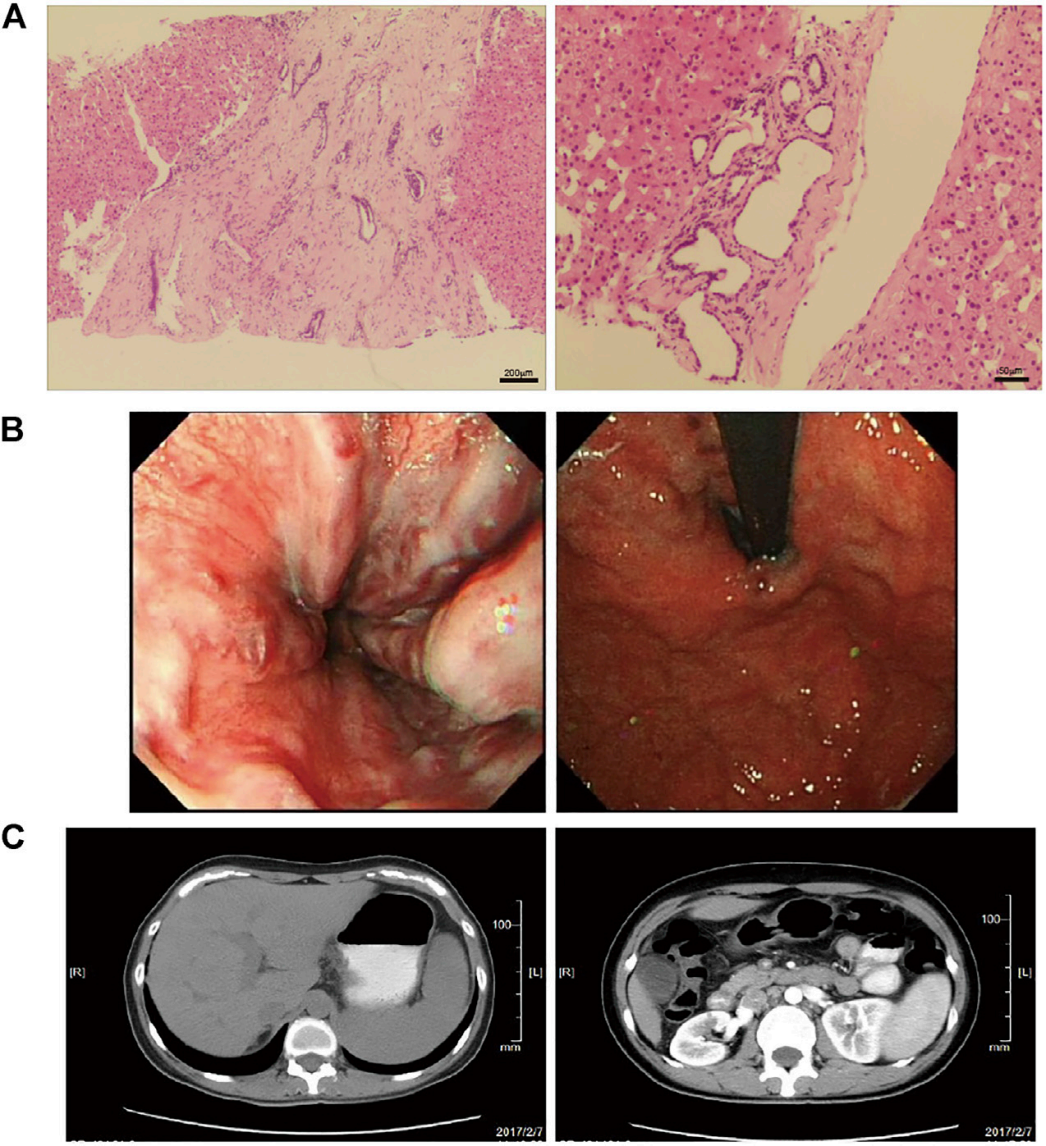
The pathological and clinical presentation of one CHF patient with NCPH. **(A)** The left panel shows diffuse fibrosis in the liver. The right panel shows a small bile duct hamartoma. **(B)** Endoscopy showed esophageal varices (left) and gastric varices (right). **(C)** The abdomen CT images show slight dilatation of the intrahepatic bile duct and splenomegaly (left), and multiple renal cysts in the bilateral kidneys (right).

The prevalence of MRC in NCPH patients accounted for 19.5% (52/267). It was significantly higher than that in cirrhotic patients with portal hypertension (6.2%, 513/8,295), *p* < 0.05. The clinical characteristics of NCPH patients with MRC were compared with 92 hepatitis B virous (HBV) related cirrhosis without MRC, which randomly selected (1:2) in cohort 1 (Table 1). The NCPH patients with MRC had a relatively older onset complications of portal hypertension (*p* < 0.05). In terms of the manifestations of portal hypertension, the proportion of EGVB was prominent (*p* < 0.05), while ascites was less (*p* < 0.001). The platelet counts were higher than that of hepatitis B cirrhosis (*p* < 0.001). Although there was no significant difference in LSM between the two groups, the ratio of platelet counts/LSM in NCPH patients with MRC was significantly higher than it in hepatitis B cirrhosis without MRC patients (*p* < 0.001). The clinical characteristics of nine patients with MRC enrolled for WGS were summarized in Table 2. Three patients (P1-3) showed CHF by liver biopsy (Figure 1A), in which two patients exhibit early-onset in childhood or adolescence and underwent splenectomy procedure before their visit to our hospital. The endoscopy and CT imaging confirmed EGV, dilated bile ducts and multiple renal cysts in those patients (Figures 1B,C).

**TABLE 1:** Comparison of clinical characteristics between NCPH patients with MRC and Hepatitis B cirrhosis without MRC.

	NCPH with MRC	Hepatitis B cirrhosis without MRC	P
	(*N* = 52)	(*N* = 92)	
Age (y)	60.35 ± 15.47	52.13 ± 10.13	0.011
Sex (female/male)	24/28	31/61	0.076
EGVB	23 (44.23%)	31 (33.70%)	0.027
Ascites	15 (28.85%)	49 (53.26%)	<0.001
Platelet (10^9^/L)	108.00 (74.50, 141.50)	56.50 (41.00, 88.00)	<0.001
INR	1.12 (1.04, 1.26)	1.23 (1.12, 1.37)	0.003
ALT (U/L)	23.65 (19.23, 36.38)	22.15 (17.45, 32.95)	0.13
AST (U/L)	37.15 (26.73, 60.45)	30.65 (23.95, 47.25)	0.308
TBIL (μmol/L)	19.85 (14.58, 41.40)	21.60 (15.83, 34.68)	0.087
ALB (g/L)	34.83 ± 6.62	34.23 ± 5.87	0.128
Creatinine (μmol/L)	67.20 (53.45, 87.88)	63.35 (53.75, 72.10)	0.133
Child-pugh score	6.00 (5.00, 8.00)	6.00 (5.00, 7.75)	0.965
MELD	5 (2, 7)	6 (3, 9)	0.559
LSM (kPa)	18.80 (13.25, 30.15)	21.80 (13.95, 30.35)	0.146
PV (mm)	13.05 ± 2.66	14.03 ± 3.15	0.637
SV (mm)	8.15 ± 1.37	9.05 ± 2.31	0.322
Platelet/LSM	5.81 ± 1.16	2.56 ± 0.57	<0.001

MRC, multiple renal cysts; EGVB, esophageal and gastric varices bleeding; ALB, Albumin; ALT, alanine aminotransferase; AST, aspartate aminotransferase; TBIL, total bilirubin; INR, international normalized ratio; MELD, model for end-stage liver disease; LSM, liver stiffness measurement; PV, portal vein diameter; SV, splenic vein diameter.

**TABLE 2:** The clinical data for nine patients with MRC enrolled for whole-genome sequencing.

	P1	P2	P3	P4	P5	P6	P7	P8	P9
Age (at first diagnose)	26 (5)	36 (34)	35 (13)	60 (55)	75 (73)	71 (60)	53 (45)	46 (36)	47 (42)
Complication of PH	EVB	EVB	No	No	No	Ascites	EVB	EVB	No
Child-Pugh Score	5	5	8	6	8	10	8	5	5
LSM (kPa)	14.3	35	15.2	5.8	8	9	12	2.8	3.8
EGV	Severe	Severe	Severe	No	Moderate	Moderate	Severe	Severe	No
Renal function	Normal	Normal	CRF II	Normal	Normal	Normal	Normal	Normal	Normal
ALT/AST	43.2/47.4	Normal	53.2/42.4	49.7/40.2	53.2/82.7	Normal	Normal	Normal	Normal
Hypersplenism	Yes	Yes	Yes	No	Yes	Yes	Yes	No	No
No. renal cysts	3	3	4	3	2	3	2	3	2
Maximum renal cyst (mm)	38	13	24	24	13	15	4	30	25
No. hepatic cysts	0	0	3	1	5	0	3	3	2
Maximum liver cyst (mm)	NA	NA	23	4	17	NA	8	7	14
Dilatation of the intrahepatic bile duct	Yes	Yes	Yes	No	No	No	No	No	No
PV (mm)	9	10	12	14	13	13	14	11	12

PH, portal hypertension; EGVs, esophageal and gastric varices; EVB, esophageal varices bleeding; LSM, liver stiffness measurement; PV, portal vein diameter; CRF: chronic renal failure; ALT, alanine aminotransferase; AST, aspartate aminotransferase. The units for ALT, and AST, was U/L. NA, no data.

The others NCPH patients (P4 and P5) showed late-onset of complication, their clinical manifestations, including large hepatorenal cysts and normal LSM, implied underlying HFDs although there is no pathological evidence. Moreover, four HBV-positive patients (P6-P9) were diagnosed at middle age with normal liver function may also pinpoint to the need of dissecting genetic factors for HFD phenotypic expression.

### Early Onset Harbored Compound Rare Pathogenic Variants in *PKHD1*

**FIGURE 2:**
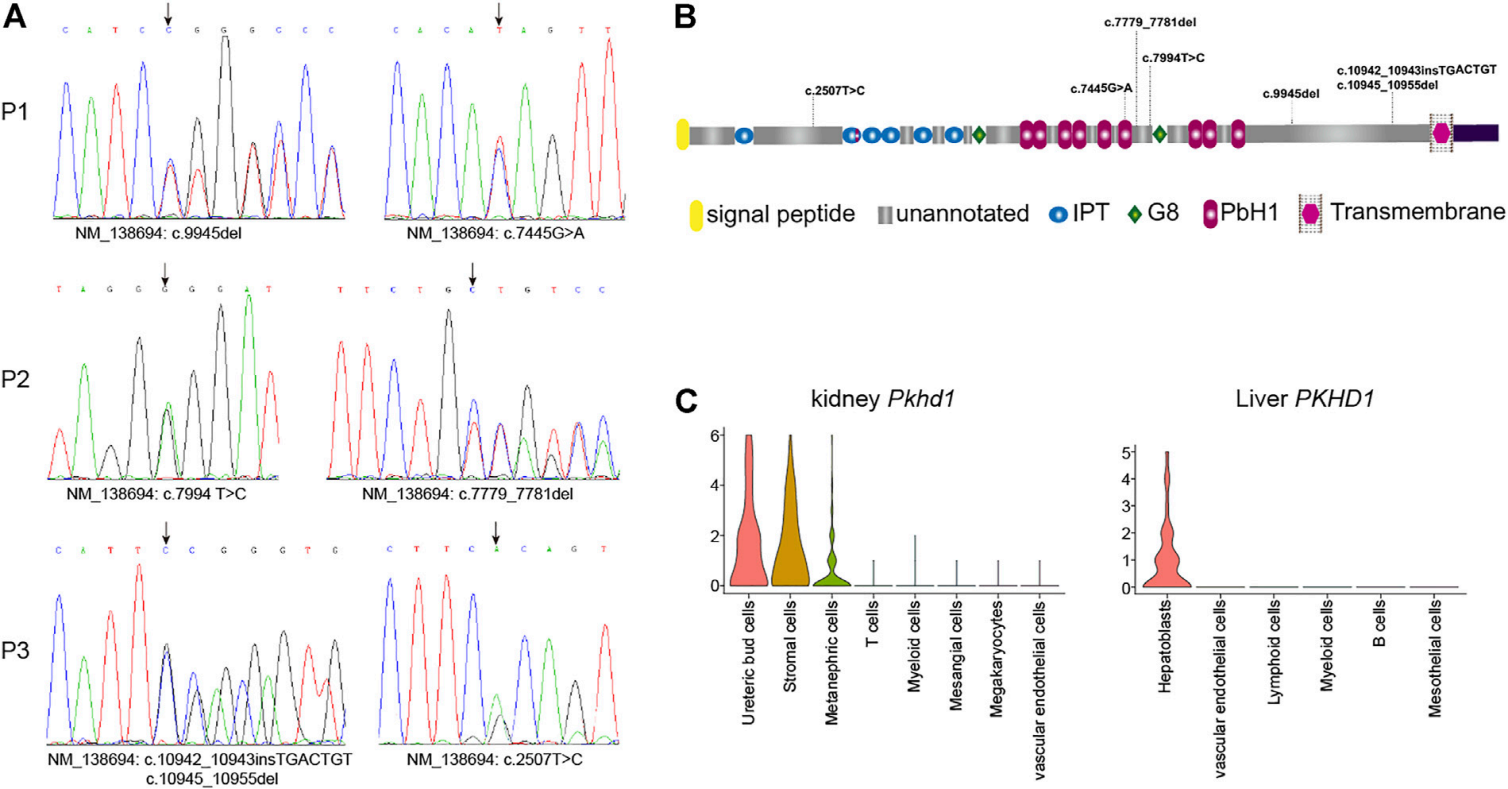
Gene mutations analysis of *PKHD1*. **(A)** The rare putative pathogenic variants in *PKHD1* were validated by Sanger sequencing. **(B)** Schematic representation of variant distributions in the *PKHD1* domain. **(C)** Violin plot of *PKHD1* expression in mouse kidney and human liver tissues by scRNA-seq analysis. The Y axis was normalized expression level for PKHD1 in different cells.

The copy number variations or structural variants spanning known HFD-related genes were not observed. Subsequently, we identified all gene mutations in the known Caroli syndrome-causing gene *PKHD*1 in 3 CHF patients with NCPH (Supplementary Table S1). All missense mutations in *PKHD1* had GERP scores higher than 5.4, indicating that these mutations were in evolutionarily highly conserved regions. Moreover, all three patients harbored an additional truncation mutation, which were not reported in any public databases. Therefore, we assumed that the patients carried recessive mutations in *PKHD1*, and validated the mutations by Sanger sequencing (Figure 2A). In addition, analysis of *PKHD1* mutation distributions in different protein domains showed that all mutations were located in the extracellular domain (Figure 2B). Further, by using scRNA-seq data to explore *PKHD1* expression, we found that *PKHD1* was highly expressed in ureteric bud cells and stromal cells and moderately expressed in metanephric cells in the kidney. In contrast, *PKHD1* was highly expressed on hepatoblasts in the liver (Figure 2C). Moreover, we also detected *PKD1* compound mutations, which may explain that the patient P1 had early disease onset at the age of five and large renal cysts (38 mm).

### Potential Candidate Genes Associated With HFDs Phenotype

**FIGURE 3:**
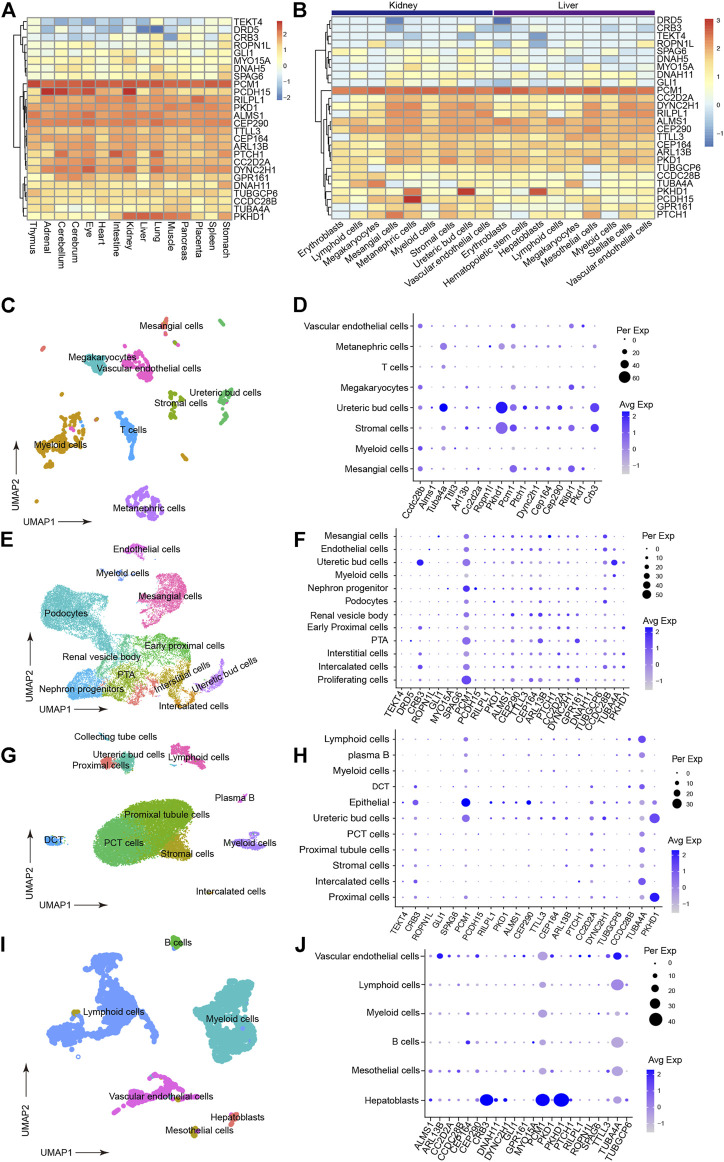
Prioritization of detected cilia genes with scRNA-seq. **(A)** Heatmap of detected cilia gene expression in multiple human fetal organs using data under accession GSE156793. Gene expression have been scaled with normalized read counts for each gene. **(B)** Heatmap of detected cilia gene expression in different cell types from human fetal kidney and liver tissues (from GSE156793). Gene expression have been scaled with normalized read counts for each gene. **(C)** UMAP plot showing the clustering of different cell types in mouse kidneys (from GSE140023). **(D)** Dot plot of cilia genes in mouse kidney (from GSE140023). The expression inferred Pkhd1, Crb3 and Tuba4a had similar expression pattern. Dot size indicated percentage of cell which expressed gene of interest and color indicated expression level. **(E)** UMAP plot showing the clustering of different cell types in human fetal kidney (from GSE114530). **(F)** Dot plot reflecting the cell type expression patterns of the detected cilia genes (from GSE114530). This result supported that PKHD1 and CRB3 had similar expression pattern. **(G)** UMAP plot showing the clustering of different cell types in human fetal kidney (from GSE131685). **(H)** Dot plot reflecting the cell type expression patterns of the detected cilia genes (from GSE131685). **(I)** UMAP plot showing the clustering of different cell types in the adult human liver (from GSE159929). **(J)** Dot plot reflecting the cell type expression patterns of the detected cilia genes (from GSE159929).

**FIGURE 4:**
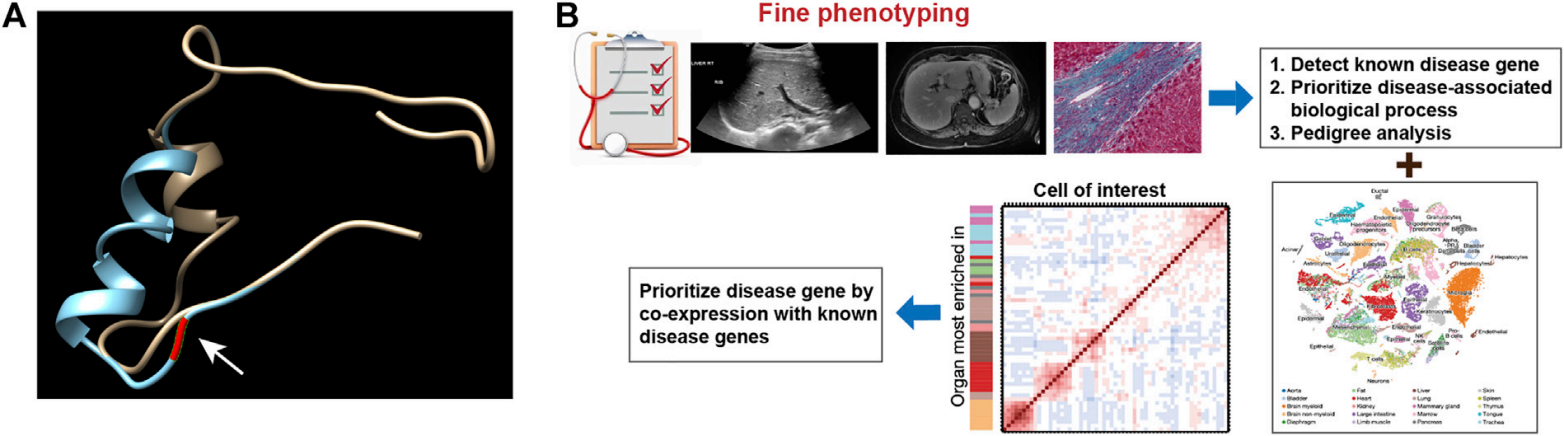
**(A)** Prediction of the conformation of CRB3 protein. The yellow chain shows the wild-type protein, and the blue chain shows the mutated protein. Residue 114 is pointed by arrow and highlighted in red on the protein backbone. **(B)** Schematic illustration of the novel analytic strategy to identify disease-related genes.

To narrow the potential HFD phenotype-associated genes, we retrieved all known cilia genes from the literature and the European project SYSCILIA (Boldt et al., 2016). After prioritizing candidate disease-associated cilia genes by scRNA-seq analysis, the cilia-related mutations derived from the enrolled patients were listed in Supplementary Table S1, such as *CRB3* p.P114L. We first plotted the expression for candidate cilia genes in multiple human fetal organs, and annotated different cell types, particularly in the kidneys and liver (Figures 3A,B). We further assessed the cilia genes of cell clustering and annotated different cell type in adult mice kidney and adult human liver using uniform manifold approximation and projection (UMAP). We found that *CRB3, TUBA4A, PTCH1* and *CEP290* co-expressed with *PKHD1* (Figures 3C,D). Similar co-expression pattern were also seen in human fetal kidney (Figures 3E,F) and adult kidney (Figures 3G,H). Followed by cell clustering and annotation in adult human liver, the *CRB3* and *PKHD1* co-expression were spotted in hepatoblasts (Figures 3I,J). To further investigate the functional effect of the *CRB3* p.P114L variants, we performed protein structure remodeling and predicted that the mutation would lead to protein conformational alterations in the PDZ-domain (Figure 4A). The bona fide tight junction assembly acquired the capacity for *PRKC1/PARD6A* complex translocation to the apical surface by interacting with the CRB3 C-terminus. Hence, we speculated that this mutation might ultimately blocking CRB3 function. With additional scRNA-seq data, we believe that this newly proposed analytical strategy may help clinicians and geneticists to map disease-related genes (Figure 4B).

## Discussions

Currently, the etiologies and genetic pathogenesis of NCPH with CHF, especially idiopathic non-cirrhotic portal hypertension (INCPH), have not been fully elucidated (Lanktree et al., 2021). The MRC is more common in CHF and NCPH (Vilarinho et al., 2016; Boëlle et al., 2019; McConnachie et al., 2021). We have reasons to believe that MRC, as a cilia, may have genetic variation disorders, especially non-classical genetic mutations of HFD phenotype in INCPH with MRC patients. In this study, the prevalence of MRC in NCPH patients was accounted almost for 19.5%, which was significantly higher than the cirrhotic portal hypertension patients with known etiologies. The clinical characteristics of NCPH with MRC from these data were older-onset of the complications of portal hypertension, obvious manifestations of portal hypertension, such as EGVB, and having preserved liver functions.

Genome-wide single-cell analysis represents the ultimate frontier of genomics research (Boldt et al., 2016). In particular, scRNA-seq studies have been boosted in the last few years of new technologies enabling the study of the transcriptomic landscape of thousands of single cells in complex pathogenesis of diseases (Ying et al., 2021). Owing to the dramatic improvement in scRNA-seq technology, especially integrating WGS and scRNA-seq, tissue-specific expression at the single-cell level has improved our understanding of biological processes (Zeggini et al., 2019). In this study, WGS and scRNA-seq were performed to survey potential genetic modifiers or candidate disease genes in NCPH patients with MRC. The results showed that genes also expressed in ureteric bud cells, stromal cells, and hepatoblasts may have additive effects on NCPH with MRC. We also found that *CRB3, TUBA4A, PTCH1* and *CEP290* co-expressed with *PKHD1* at hepatoblasts in liver using UMAP. Interestingly, we discovered that patient P5 carried a homozygous candidate mutation in *CRB3* without family history of MRC. The *CRB3* encodes an apical transmembrane protein that regulates the morphogenesis of tight junctions in mammalian epithelial cells (Lemmers et al., 2004). CRB3 protein plays an important role in apicobasal polarity formation, such as cyst formation (Hurd et al., 2003). Furthermore, CRB3 participates in interactions with *TAZ/YAP*, thereby affecting transforming growth factor (TGF)-β signaling. Disruption of CRB3 function enhances TGF-β signaling and predisposes cells to TGF-β-mediated epithelial-to-mesenchymal transition (Varelas et al., 2010). Therefore, loss of function of CRB3 could potentially be linked to cyst formation and/or fibrosis. Importantly, further narrowing of the candidate gene selection showed that *CRB3* could be a novel disease risk gene for HFDs. Although patients carrying a homozygous mutation in *CRB3* showed late disease onset, this mutation affects PDZ domain conformation and might alleviate protein function rather than cause complete loss of function. However, further studies are needed for functional validation of the pathogenicity of this gene.

One of the limitations of this study is the lack of parental genomic materials and family pedigree of MRC, making it challenging to further prioritize selected candidates. In addition, the present study lacks WGS analysis in MRC patients with mutations in known pathogenic genes, such as ARPKD phenotypes associated genes and *PKHD1* or *PKD* genes because of small sample size of NCPH with MRC and hepatitis B cirrhosis patients without MRC. Furthermore, the novel identified *CRB3* p.P114L variant has not been undergone biological function study, and we will conduct the research in the future.

## Conclusion

*CRB3* gene is commonly co-expressed with *PKHD1* in NCPH with MRC. The homozygous variant in *CRB3* may be associated with genetic pathogenesis of NCPH with MRC. Therefore, we speculate that there may be non-classical genetic mutations in NCPH patients with MRC. *CRB3* may be a novel homozygous candidate gene mutation.

## Data Availability

The datasets presented in this study can be found in online repositories. The names of the repository/repositories and accession number(s) can be found in the article/Supplementary Material.
