# Supplementary material for: Identification of Genetic Predisposition in Noncirrhotic Portal Hypertension Patients With Multiple Renal Cysts by Integrated Analysis of Whole-Genome and Single-Cell RNA Sequencing
Source: Front Genet. 2021 Nov 12;12:775470. doi: 10.3389/fgene.2021.775470 (PMC8633307; doi:10.3389/fgene.2021.775470)
Supplement: Supplementary file 3 [file Table1.DOC]

Supplementary Material

**Supplement table1**. **The rare putative genetic variants in cilia genes were detected in 9 patients**

| Gene | Transcript | Type | cDNA change | Protein change | MAF | P1 | P2 | P3 | P4 | P5 | P6 | P7 | P8 | P9 |
| --- | --- | --- | --- | --- | --- | --- | --- | --- | --- | --- | --- | --- | --- | --- |
| CCDC28B | NM_024296 | missense | c.263T>C | p.V88A | NA | 0/0 | 0/0 | 0/0 | 0/0 | 0/0 | 0/0 | 0/0 | 0/0 | 0/1 |
| GPR161 | NM_001267611 | missense | c.68C>G | p.S23C | NA | 0/0 | 0/0 | 0/0 | 0/0 | 0/0 | 0/0 | 0/0 | 0/1 | 0/0 |
| ALMS | NM_015120 | Indel | c.41_42insGGA | p.E14insE | NA | 0/0 | 0/0 | 0/0 | 0/1 | 0/0 | 0/0 | 0/0 | 0/0 | 0/0 |
| TEKT4 | NM_144705 | splicing | c.713+2T>G | No data | 0.0028 | 0/1 | 0/0 | 0/0 | 0/0 | 0/0 | 0/0 | 0/0 | 0/0 | 0/1 |
| TEKT4 | NM_144705 | missense | c.1031G>T | p.R344L | 3.25E-05 | 0/0 | 0/0 | 0/0 | 0/0 | 0/0 | 0/0 | 0/0 | 0/0 | 0/1 |
| TUBA4A | NM_006000 | missense | c.125T>C | p.I42T | 0.0021 | 0/0 | 0/0 | 0/0 | 0/1 | 0/0 | 0/0 | 0/0 | 0/0 | 0/0 |
| TTLL3 | NM_001025930 | indel | c.2446_2450del | p.R816fs | NA | 0/0 | 0/1 | 0/0 | 0/0 | 0/0 | 0/0 | 0/0 | 0/0 | 0/0 |
| ARL13B | NM_144996 | missense | c.188G>C | p.G63A | 5.8E-05 | 0/0 | 0/0 | 0/0 | 0/0 | 0/0 | 0/0 | 0/0 | 0/0 | 0/1 |
| DRD5 | NM_000798 | indel | c.865_867del | p.289_289del | 0.001 | 0/0 | 0/0 | 0/0 | 0/0 | 0/0 | 0/0 | 0/0 | 0/1 | 0/0 |
| CC2D2A | NM_001080522 | missense | c.2944C>T | p.R982C | 0.0002 | 0/0 | 0/0 | 0/0 | 0/1 | 0/0 | 0/0 | 0/0 | 0/0 | 0/0 |
| ROPN1L | NM_001201466 | Splicing in 5’-UTR | | No data | 0.0006 | 0/0 | 0/0 | 0/0 | 0/0 | 0/0 | 0/1 | 0/0 | 0/0 | 0/0 |
| DNAH5 | NM_001369 | missense | c.2047C>T | p.R683W | 0.0062 | 0/0 | 0/0 | 0/0 | 0/0 | 0/0 | 0/0 | 0/0 | 0/1 | 0/0 |
| DNAH5 | NM_001369 | missense | c.892A>G | p.N298D | 0.0008 | 0/0 | 0/0 | 0/0 | 0/0 | 0/1 | 0/0 | 0/0 | 0/0 | 0/0 |
| DNAH5 | NM_001369 | missense | c.817C>G | p.Q273E | NA | 0/0 | 0/0 | 0/1 | 0/0 | 0/0 | 0/0 | 0/0 | 0/0 | 0/0 |
| PKHD1 | NM_138694 | indel | c.10945_10955del | p.A3649fs | NA | 0/0 | 0/0 | 0/1 | 0/0 | 0/0 | 0/0 | 0/0 | 0/0 | 0/0 |
| PKHD1 | NM_138694 | indel | c.10942_10943ins  TGACTGTT | p.R3648fs | NA | 0/0 | 0/0 | 0/1 | 0/0 | 0/0 | 0/0 | 0/0 | 0/0 | 0/0 |
| PKHD1 | NM_138694 | indel | c.9945delG | p.R3315fs | NA | 0/1 | 0/0 | 0/0 | 0/0 | 0/0 | 0/0 | 0/0 | 0/0 | 0/0 |
| PKHD1 | NM_138694 | missense | c.7994T>C | p.L2665P | NA | 0/0 | 0/1 | 0/0 | 0/0 | 0/0 | 0/0 | 0/0 | 0/0 | 0/0 |
| PKHD1 | NM_138694 | indel | c.7779_7781del | p.2593_2594del | NA | 0/0 | 0/1 | 0/0 | 0/0 | 0/0 | 0/0 | 0/0 | 0/0 | 0/0 |
| PKHD1 | NM_138694 | missense | c.7445G>A | p.C2482Y | 0.0003 | 0/1 | 0/0 | 0/0 | 0/0 | 0/0 | 0/0 | 0/0 | 0/0 | 0/0 |
| PKHD1 | NM_138694 | missense | c.2507T>C | p.V836A | 0.0013 | 0/0 | 0/0 | 0/1 | 0/0 | 0/0 | 0/0 | 0/0 | 0/0 | 0/0 |
| DNAH11 | NM_001277115 | missense | c.8275T>C | p.F2759L | 0.0031 | 0/1 | 0/0 | 0/0 | 0/0 | 0/0 | 0/0 | 0/0 | 0/0 | 0/1 |
| DNAH11 | NM_001277115 | missense | c.9166A>G | p.S3056G | 0.0001 | 0/0 | 0/0 | 0/0 | 0/0 | 0/0 | 0/1 | 0/0 | 0/0 | 0/0 |
| DNAH11 | NM_001277115 | missense | c.12428T>C | p.M4143T | 0.001 | 0/0 | 0/0 | 0/0 | 0/1 | 0/0 | 0/0 | 0/0 | 0/0 | 0/0 |
| PCM1 | NM_006197 | missense | c.3452A>G | p.D1151G | NA | 0/0 | 0/0 | 0/1 | 0/0 | 0/0 | 0/0 | 0/0 | 0/0 | 0/0 |
| PTCH1 | NM_000264 | missense | c.2320G>A | p.G774R | NA | 0/0 | 0/0 | 0/0 | 0/0 | 0/0 | 0/0 | 0/1 | 0/0 | 0/0 |
| SPAG6 | NM_012443 | missense | c.31G>C | p.E11Q | 0.0055 | 0/0 | 0/0 | 0/0 | 0/1 | 0/0 | 0/0 | 0/0 | 0/0 | 0/0 |
| PCDH15 | NM_033056 | indel | c.4322_4323ins  GCCGCC | p.P1441_I1442  delinsPPP | 0.0074 | 0/1 | 0/0 | 0/0 | 0/0 | 0/0 | 0/0 | 0/0 | 0/0 | 0/0 |
| DYNC2H1 | NM_001377 | missense | c.11048C>A | p.P3683Q | NA | 0/0 | 0/0 | 0/0 | 0/0 | 0/0 | 0/1 | 0/0 | 0/0 | 0/0 |
| CEP164 | NM_014956 | missense | c.59A>C | p.E20A | 0.0006 | 0/0 | 0/0 | 0/0 | 0/0 | 0/0 | 0/1 | 0/0 | 0/0 | 0/0 |
| GLI1 | NM_005269 | stopgain | c.337C>T | p.R113X | 3.25E-05 | 0/0 | 0/0 | 0/0 | 0/1 | 0/0 | 0/0 | 0/0 | 0/0 | 0/0 |
| CEP290 | NM_025114 | missense | c.1874G>T | p.R625M | 0.0001 | 0/1 | 0/0 | 0/0 | 0/0 | 0/0 | 0/0 | 0/0 | 0/0 | 0/0 |
| RILPL1 | NM_178314 | stopgain | c.1132G>T | p.E378X | 0.001 | 0/0 | 0/1 | 0/0 | 0/0 | 0/0 | 0/0 | 0/0 | 0/0 | 0/0 |
| PKD1 | NM_000296 | missense | c.7096G>T | p.V2366L | NA | 0/1 | 0/0 | 0/0 | 0/0 | 0/0 | 0/0 | 0/0 | 0/0 | 0/0 |
| PKD1 | NM_000296 | missense | c.6285C>A | p.D2095E | 0.0012 | 0/1 | 0/0 | 0/0 | 0/0 | 0/0 | 0/0 | 0/0 | 0/0 | 0/0 |
| MYO15A | NM_016239 | missense | c.2522C>T | p.P841L | NA | 0/0 | 0/1 | 0/0 | 0/0 | 0/0 | 0/0 | 0/0 | 0/0 | 0/0 |
| CRB3 | NM_13916 | missense | c.341C>T | p.P114L | 0.0006 | 0/0 | 0/0 | 0/0 | 0/0 | 1/1 | 0/0 | 0/0 | 0/0 | 0/0 |
| TUBGCP6 | NM_020461 | missense | c.5426G>A | p.R1809H | 0.0011 | 0/0 | 0/0 | 0/0 | 0/0 | 0/0 | 0/1 | 0/0 | 0/0 | 0/0 |
| TUBGCP6 | NM_020461 | missense | c.3676G>A | p.G1226R | 0.0001 | 0/0 | 0/1 | 0/0 | 0/0 | 0/0 | 0/0 | 0/0 | 0/0 | 0/0 |

The last nine column indicated the genotype: 0/1 stand for heterozygous and 1/1 stand for homozygous, MAF: Minor allele frequency, the highest MAF in 1000G and gnomAD ethnic group was listed. NA: not available.
